# The Tobii Pro Spectrum: A useful tool for studying microsaccades?

**DOI:** 10.3758/s13428-020-01430-3

**Published:** 2020-07-23

**Authors:** Marcus Nyström, Diederick C. Niehorster, Richard Andersson, Ignace Hooge

**Affiliations:** 1 Lund University Humanities Lab, Box 201, SE–221 00 Lund, Sweden; 2 Department of Psychology, Lund University Humanities Lab, Box 201, SE–221 00 Lund, Sweden; 3 Tobii Pro AB, Box 743, SE–182 17 Danderyd, Sweden; 4 Experimental Psychology, Helmholtz Institute, Utrecht University, Heidelberglaan 1, 3584 CS Utrecht, The Netherlands

**Keywords:** Microsaccades, EyeLink 1000 Plus, Tobii Pro Spectrum

## Abstract

Due to its reported high sampling frequency and precision, the Tobii Pro Spectrum is of potential interest to researchers who want to study small eye movements during fixation. We test how suitable the Tobii Pro Spectrum is for research on microsaccades by computing data-quality measures and common properties of microsaccades and comparing these to the currently most used system in this field: the EyeLink 1000 Plus. Results show that the EyeLink data provide higher RMS precision and microsaccade rates compared with data acquired with the Tobii Pro Spectrum. However, both systems provide microsaccades with similar directions and shapes, as well as rates consistent with previous literature. Data acquired at 1200 Hz with the Tobii Pro Spectrum provide results that are more similar to the EyeLink, compared to data acquired at 600 Hz. We conclude that the Tobii Pro Spectrum is a useful tool for researchers investigating microsaccades.

## Introduction

Fixational eye movements—i.e., those that humans and some animals produce when they have the task to fixate on an object—consist of small (micro) saccades, slow drift, and high-frequency, low-amplitude tremor (Martinez-Conde et al., 2004; Rolfs, 2009; Rucci & Victor, 2015). Despite early work dismissing fixational eye movements as oculomotor noise or even evolutionary mistakes (Kowler & Steinman, 1980), there has over the past two decades been a surge in work on fixational eye movements, which have been linked to various perceptual and cognitive functions, and connected to neurological underpinnings (Martinez-Conde et al., 2013). For example, the occurrence of microsaccades has been linked with the allocation of covert attention (Engbert & Kliegl, 2003), onset of visual (Scholes et al., 2015) and neural (Martinez-Conde et al., 2000) transients, anticipation (Fried et al., 2014), mental fatigue (Di Stasi et al., 2013), and cognitive workload (Siegenthaler et al., 2014). It is a longstanding fact that microsaccades occur in most participants at a rate of about 1–2 Hz. However, other properties of microsaccades are still debated (Collewijn & Kowler, 2008; (Nyström et al., 2016), 2017). In the early studies in the 1950s and 1960s, for example, the agreed-upon upper limit of microsaccade amplitudes was 12 min arc (Collewijn & Kowler, 2008), whereas today 60 min arc (1 deg) is a common cut-off when distinguishing micro- from larger saccades (Martinez-Conde et al., 2013). Nyström et al. (2016) conclude that while we still do not have a definite answer to why such discrepancies in amplitudes exist between the old and the new literature, the technology to record fixational eye movements, which has changed fundamentally since the 1950s, is an important factor (for a comprehensive review, see Poletti and Rucci 2016).

Over the past decades, the EyeLink family of eye trackers is unarguably the most used eye tracker in microsaccade research. For instance, in their review of microsaccade research between 2004 and 2009, Martinez-Conde et al., (2009) list 37 studies (with 43 experiments in total) in their Table 1 of which 30 use an EyeLink. From 2010 to 2018, a Google Scholar search ends up with more hits when entering EyeLink together with microsaccade and eye tracker compared to combining the last two words with any of the other competing companies or techniques. Importantly, similar microsaccade rates were found in EyeLink 1000 data when compared to co-recorded data acquired with scleral search coils (McCamy et al., 2015), considered the ‘gold standard’ in oculomotor research (Collewijn, 1999) Today, the EyeLink 1000 Plus is the most commonly used eye tracker for microsaccade research. It is based on the traditional pupil and corneal reflection (CR) principle, where gaze locations on a calibration plane (usually a screen) are inferred from pupil-CR vectors through polynomial mapping (SR Research, 2017).

**Table 1:** Output of the linear mixed effects models predicting RMS precision, SD precision, microsaccade amplitude (Amp), and microsaccade rate (Rate). The two latter variables are generated from the algorithm by Engbert and Kliegl (2003) using a fixed *λ* = 6. The intercept corresponds to the EyeLink F setup. *Values in parenthesis* represent standard errors

	Prec (RMS, deg)	Prec (SD, deg)	Amp (deg)	Rate (Hz)
(Intercept)	0.01^∗∗^	0.23^∗∗∗^	0.29^∗∗∗^	1.43^∗∗∗^
	(0.00)	(0.02)	(0.02)	(0.15)
EyeLink U	0.02^∗∗∗^	− 0.02^∗^	0.01	0.07
	(0.00)	(0.01)	(0.01)	(0.05)
Spectrum 1200	0.05^∗∗∗^	− 0.07^∗∗∗^	− 0.02^∗^	− 0.12^∗^
	(0.00)	(0.01)	(0.01)	(0.05)
Spectrum 600	0.05^∗∗∗^	− 0.06^∗∗∗^	− 0.02^∗∗^	− 0.27^∗∗∗^
	(0.00)	(0.01)	(0.01)	(0.05)
AIC	− 8771.12	− 1879.28	− 1122.53	557.94
BIC	− 8737.80	− 1845.95	− 1097.59	582.88
Log Likelihood	4391.56	945.64	567.27	− 272.97
Num. obs.	1908	1908	472	472
Num. groups: pid	12	12	12	12
Var: pid (Intercept)	0.00	0.00	0.01	0.25
Var: Residual	0.00	0.02	0.00	0.16

^∗∗∗^*p* < 0.001, ^∗∗^*p* < 0.01, ^∗^*p* < 0.05

While the EyeLink family of eye trackers has long been the clear choice for microsaccade researchers due to its high sampling frequency and precision, other eye trackers are now approaching similar specifications. Two examples using stereo cameras and more than one source of illumination in combination with physical 3D models of the eye are the open-source eye tracker by Barsingerhorn et al., (2018) and the commercially available Tobii Pro Spectrum. These eye trackers may therefore be interesting for researchers studying fixational eye movements. However, since history has taught us that introducing a new way of measuring the small fixational eye movements also may change their measured properties, a validation of its capabilities against current instruments is crucial (Collewijn & Kowler, 2008; Nyström et al., 2016).

What is important for an eye tracker that will be used to record fixational eye movements? Since it is often not critical to know the exact position where participants are looking, small inaccuracies (systematic errors) in the eye-tracker signal are usually not problematic in the majority of research on fixational eye movements (Poletti et al., 2013, but see, for instance). Since microsaccades can be very small (Poletti and Rucci, 2016, use a lower bound of 3’) it is however critical to record data with high precision, i.e., data with a low variable error. Articles reporting data collected by an EyeLink often point the reader to the EyeLink manual, which lists a ‘spatial resolution’ of 0.01 deg (0.6’) (SR Research, 2017, p. 9), which refers to a measurement with a static artificial eye. This value is computed as the root mean square (RMS) of distances between consecutive samples.^1^ Reporting values from a manual is unfortunate since precision differs for real eyes and artificial eyes (Holmqvist et al., 2011, p. 35), and across studies depending on factors such as the eye physiology of the participants, the recording environment, the setup and settings of the eye tracker, and the method to compute precision (Nyström et al., 2013a).

A few researchers report the actual precision in the data, how to calculate it, and the state of the filters during recording. For instance, Nyström et al., (2017) reported both filtered and unfiltered standard deviation (SD) and root mean square (RMS) of intersample distances. Unfiltered data gave precision values between 0.03 to 0.06 deg, while filtered data (with a Savitzky–Golay filter of length 21 ms) provided an order of magnitude higher precision (0.003 to 0.006 deg).

Another important property is sampling frequency. Since the smallest microsaccades also have very short durations (a few milliseconds), it is critical to have a high enough sampling frequency to be able to detect them, but also to quantify more detailed properties like velocity and shape. Since frequencies above 100 Hz carry little information about fixational eye movements (Findlay, 1971), it would according to the Nyquist–Shannon theorem (Nyquist, 1928; Shannon, 1949) be sufficient to record at twice that frequency (200 Hz) to be able to capture all fixational eye movements. In practice, however, studies investigating microsaccades typically use eye trackers recording at 250 Hz and above (Martinez-Conde et al., 2009). The lower bound of 250 Hz likely reflects the fact that the SMI EyeLink I, which was the state-of-the-art video-based eye tracker in the early 2000s, had a maximum sample rate of 250 Hz.

The goal of this paper is to test whether a remote stereo-camera-based eye tracker, the Tobii Pro Spectrum, is suitable for studying microsaccades in a typical experimental paradigm where participants fixate centrally located targets on a computer screen. Suitability is defined in terms of properties of both the eye tracker signal (precision, power spectral density) and the detected microsaccades (rate, amplitude, displacement, direction, and shape). Results will be compared against one of the currently most used eye trackers in microsaccade research, the EyeLink 1000 Plus in desktop mount, across participants who perform the same task. Since recordings are performed within participants across eye trackers, we assume that any differences we find are attributed to differences between the eye trackers.

In Experiment I, four experienced participants are recorded in a fixation task where blink-free data on fixational eye movements are acquired and analyzed in detail on an individual level. To test whether the results generalize to a wider population with non-expert participants, eight naive participants are recorded in Experiment II using a less demanding fixation task. Finally, statistical analyses using all participants from both experiments are used to compare the data quality and properties of the detected microsaccades across the eye trackers.

## Experiment I

### Methods

#### Participants and apparatus

Binocular eye movements from four male participants (P1, P2, P3, P4) were recorded on the EyeLink 1000 Plus in Desktop Mode (Host version 5.12) and the Tobii Pro Spectrum (firmware version 1.7.6). To reduce the variance across setups and recordings, all of the participants were authors trained to sit very still and to avoid blinking. Each of the participants has over 10 years of experience working with eye trackers in various contexts. Two of the participants (P3, P4) wore glasses. Informed consent was obtained from each participant.

The EyeLink was set to record binocular eye movements at 1000 Hz in pupil centroid mode, and was set up according to the recommendations of the manufacturer (SR Research, 2017).

**Fig. 1:**
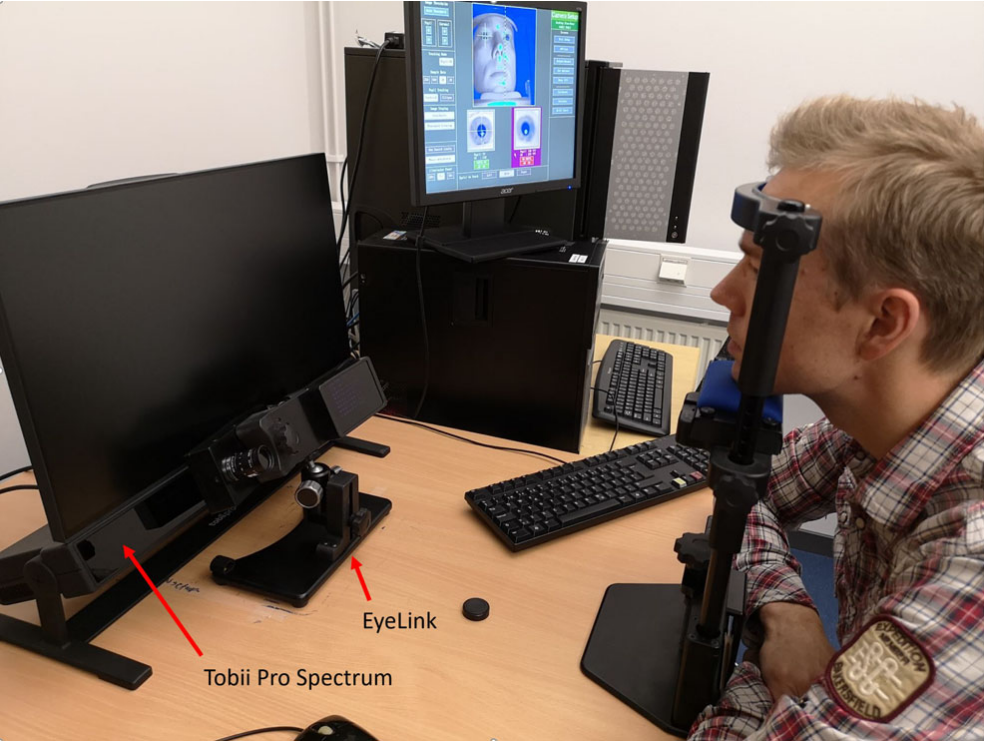
The experimental setup. To center the eyes in the track box, the Tobii Pro Spectrum was positioned 63 cm from the participant. During EyeLink recordings, the EyeLink camera was placed in front of the Spectrum at the recommended distance (52 cm)

Stimuli were presented on the native Tobii Pro Spectrum screen (EIZO FlexScan EV2451) with a resolution of 1920 × 1080 pixels (52.8 × 29.7 cm). Participants sat at a distance of 63 cm from the screen and positioned themselves such that the average position of the eyes was in the center of the headbox of the Tobii Pro Spectrum. Participants’ heads were supported with the EyeLink chin-, and forehead rest. The setup can be seen in Fig. 1.

Stimuli were presented on the screen with PsychoPy 1.85.0 Peirce (2007, 2008).

#### Procedure

Participants completed a calibration followed by a validation. For the Tobii Pro Spectrum, the default calibration in Titta (Niehorster et al., 2019) was used, where five calibration points were followed by four points for validation. The EyeLink was calibrated with the standard nine-point calibration followed by a nine-point validation. Although accuracy is not the main variable of interest in this study, re-calibrations were performed if visual inspection of the validation data revealed that there were large deviations in one or more validation points.

Prior to the onset of calibration, eye images were inspected to ensure that relevant eye features (pupil and corneal reflection(s)) were clearly visible to the operator. Prior to calibrating the EyeLink, the focus of the camera was adjusted such that the sizes of the CRs were minimized in the eye image, followed by auto-adjustments of the pupil-, and CR thresholds.

Following a calibration, each participant was recorded in four setups:

A. Tobii Pro Spectrum at 1200 Hz.
B. Tobii Pro Spectrum at 600 Hz.
C. EyeLink with both heuristic filters switched on.
D. EyeLink with both heuristic filters switched off.

Consequently, each eye tracker was tested in two setups. For the Tobii Pro Spectrum, the two highest sampling frequencies were used to investigate whether there is a sampling frequency/noise trade-off leading to a noisier signal in 1200-Hz recordings compared to 600-Hz recordings. This is to be expected due to the shorter exposure time, and hence worse image quality, in the 1200-Hz case (see Appendix A). Conversely, the higher sampling frequency may provide more information about the microsaccades, aiding their detection. Some microsaccade researchers using the EyeLink record data with the proprietary heuristic filters turned on, or do not report the state of the filter at all. Therefore we collected filtered and unfiltered data. Using unfiltered EyeLink data also makes comparisons with Tobii Pro Spectrum data (which are unfiltered) more relevant from an evaluation perspective.

In the remainder of the text, the setups will be referred to as Spectrum 1200 (A), Spectrum 600 (B), EyeLink F (C), and EyeLink U (D). For each setup, participants were asked to fixate a stimulus dot (inner diameter 0.1 deg, outer diameter 0.6 deg, denoted ABC in Thaler et al., 2013) on a mid-gray background (RGB: (128, 128, 128), luminance 37.6 cd/m^2^) in the center of the screen for five trials of 20 s each.

If the reported pupil size in either of the eyes was ‘not a number’, nan (Tobii), or less than 100 pupil area units (EyeLink), the trial was interrupted and a new trial was added to the experiment. This typically happens when a participant blinks, and was added to ensure that five 20-s trials without data loss were recorded for each participant and setup.

Each participant was recorded twice for each setup in the order ABCDABCD for two of the participants and CDABCD AB for the other two. All data for each participant were collected within one hour with only short breaks in-between.

No attempts were done during the recordings to change, e.g., in the EyeLink case, pupil thresholds in case the signal for some reason was lost.

### Data analysis

Accuracy values from the EyeLink were taken directly from the output files generated by the edf-converter tool provided by SR Research. For the Spectrum, accuracy values for each validation point were computed by extracting data in a 500-ms interval starting 500 ms after each validation point onset. The average distance between each validation point and the median value of the corresponding gaze data was taken as the accuracy value.

Prior to being fed in the microsaccade detection algorithms, data were converted to degrees and lowpass filtered with a Bartlett window filter of size of 20 ms (cf. Appendix C).

Microsaccades were detected with a standard algorithm in the field (Engbert and Kliegl, 2003), using a minimum microsaccade duration of 5 ms and *λ* = 6. To prevent counting overshoots as additional microsaccades, an additional requirement was that a minimum duration of 10 ms had to separate two consecutive microsaccades. Microsaccade rate was computed per trial by dividing the number of microsaccades with the total duration of valid samples in the trial; this to prevent that, for instance, trials with few microsaccades and much data loss would be taken as a low microsaccade rate of a participant.

To investigate how the choice of detection algorithm influences the results (cf. Appendix D), microsaccades were also detected with the algorithm by Otero-Millan et al., (2014) and, following Engbert and Mergenthaler (2006), by using surrogate data to find optimal *λ* values for the Engbert & Kliegl-algorithm.

### Results

#### Data quality

**Fig. 2:**
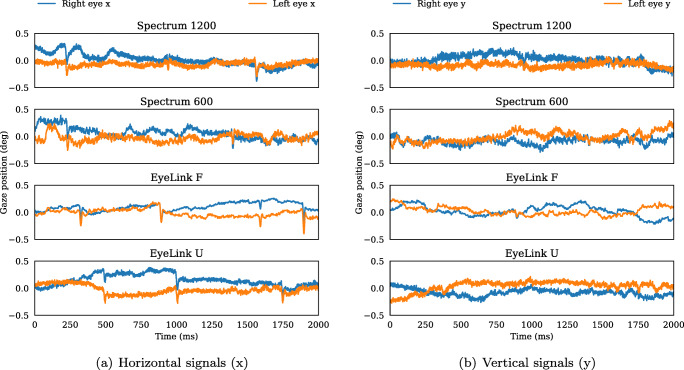
Horizontal (**a**) and vertical (**b**) gaze signals from the left and the right eyes for a 2-s-long period. The data were collected from the same participant on two different eye trackers with two different settings. The plot titles refer to Tobii Pro Spectrum at 1200 Hz (Spectrum 1200), Tobii Pro Spectrum at 600 Hz (Spectrum 600), EyeLink at 1000 Hz with both heuristic filters switched on (EyeLink F), and EyeLink at 1000 Hz with both heuristic filters switched off (EyeLink U)

Average accuracy over all validation points, participants, and eyes was (in degrees): EyeLink F (M = 0.44, SD = 0.28), EyeLink U (M = 0.56, SD = 1.02), Spectrum 1200 (M = 0.58, SD = 0.38, Spectrum 600 (M = 0.58, SD = 0.37). Figure 2 shows two seconds of binocular horizontal and vertical gaze signals recorded from one participant in the four recording setups. Since microsaccades occur at rates of about 1–2 Hz and are more prevalent in the horizontal direction (Rolfs, 2009), we expect to see at least a few examples of microsaccades in the horizontal component of the signals. Indeed, microsaccades can clearly be seen with the naked eye in all four setups in Fig. 2a. In contrast, microsaccades are virtually absent in the vertical data (Fig. 2b). From visual inspection, the signal-to-noise ratio appears to be similar in the first, second, and fourth panels. Unsurprisingly, EyeLink data are less noisy when switching on the heuristic filters (third panel).

**Fig. 3:**
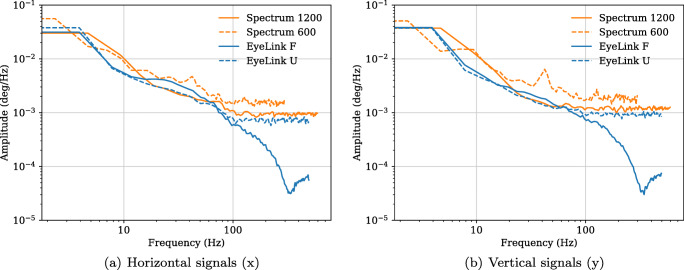
Power spectral density (PSD) for (**a**) horizontal and (**b**) vertical signals from one representative participant and trial. The plots were generated with the psd function in matplotlib (v. 2.1.2) using default settings. Each signal ends at half the sampling frequency of the tested setup

To characterize the noise, the power spectral density (PSD) of the signals was computed using Python 2.7 and the psd function in matplotlib (v. 2.1.2, default settings). In short, the PSD is a measure of the strength (power) of the variations in a signal as a function of frequency. It is computed over 256 sample long windows by calculating the average of the Fourier transform of each window. Figure 3 illustrates how the power of signals from the four setups is distributed across different frequencies for a representative trial.

There are a few observations one can make from this figure. First, the EyeLink data recorded with the heuristic filters turned on have a very different distribution of power at frequencies above 100 Hz. Instead of a flat power spectrum, indicating white noise, the power of the filtered data reaches a local minimum between 300 and 400 Hz, after which it starts to increase again. Second, since noise from a completely still artificial eye typically is white and thus flat (Coey et al., 2012; Wang et al., 2017), it is likely that eye movements, which are characterized by pink (1/f) noise (Coey et al., 2012), contribute with power only at frequencies lower than 100 Hz (*cf.* Findlay, 1971). Since the power at a certain frequency is proportional to the squared amplitude of the signal, it can from the higher frequency range (> 100 Hz) in this plot also be predicted that the precision of the gaze signals from the recording setups will be ordered in the same way as in Fig. 3, i.e., highest to lowest: EyeLink F, EyeLink U, Spectrum 1200, Spectrum 600.

**Fig. 4:**
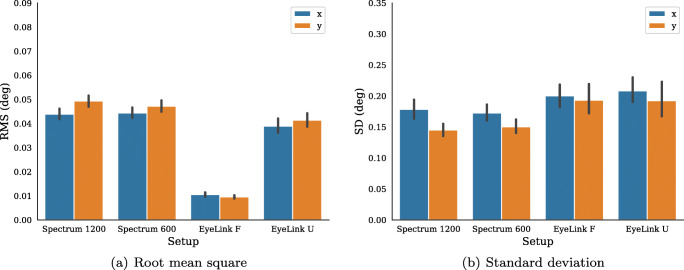
Root mean square (RMS) of inter-sample distances (**a**) and standard deviation (SD) (**b**) of gaze signals for each recording setup. *Error bars* show 95% confidence intervals of the mean; x and y refer to the horizontal and vertical dimensions of the data

To quantify precision, the root-mean-square (RMS) of sample-to-sample distances and the standard deviation (SD) of the signals are computed for all participants across each trial (Fig. 4). SD was included to capture slower variations in the signal indicative of, e.g., drift. Overall, the above observations were confirmed, and the horizontal and vertical RMS of the unfiltered EyeLink data (EyeLink U) had marginally lower values than that of the Spectrum recordings.

Horizontal and vertical SD is on average higher in the EyeLink data compared to the Spectrum data, and only small differences exist between filtered and unfiltered EyeLink data.

Detailed summary statistics of precision values for each participant and measurement is provided in Table 4, which shows that precision appears to be stable within participants and across recordings.

#### Microsaccades

While the calculated precision of data can be improved by lowpass filters internal or external to the eye tracker, high precision *per se* does not mean that microsaccades can be measured and represented more accurately. Therefore, a detailed analysis of detected microsaccades and their properties follows below.

Since experienced participants were recorded within a limited time period while performing the same task, we assume a similar microsaccade production within participants and across setups.

**Fig. 5:**
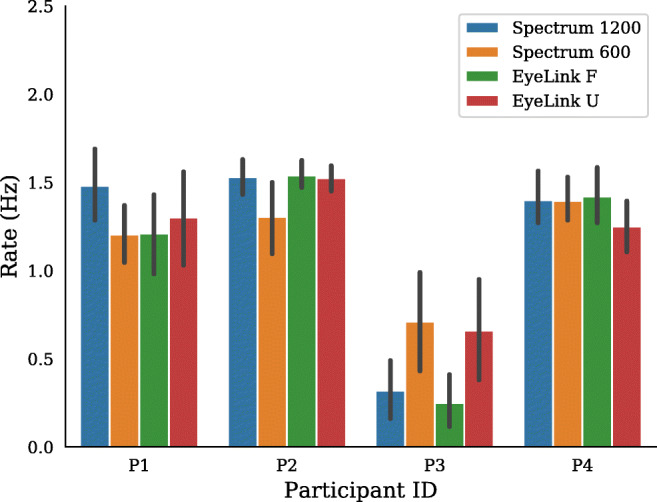
Experiment I. Microsaccades rates across the four setups for all four participants. *Error bars* show 95% confidence intervals

Microsaccade rates for each participant and setup are shown in Fig. 5. The data show some variation across participants and setups, but it does not seem like a particular setup systematically shows lower or higher microsaccade rates.

**Fig. 6:**
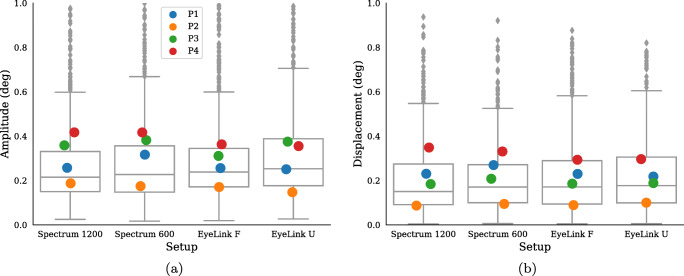
Microsaccades amplitude (**a**) and displacement (**b**) across the four setups for all expert participants in Experiment I. The *boxes* show the median value along with the upper and lower quartiles and the *whiskers* extend 1.5 times the inter-quartile range, and values outside this range are represented as *diamonds*. The *Colored dots* represent each participant’s mean value

**Fig. 7:**
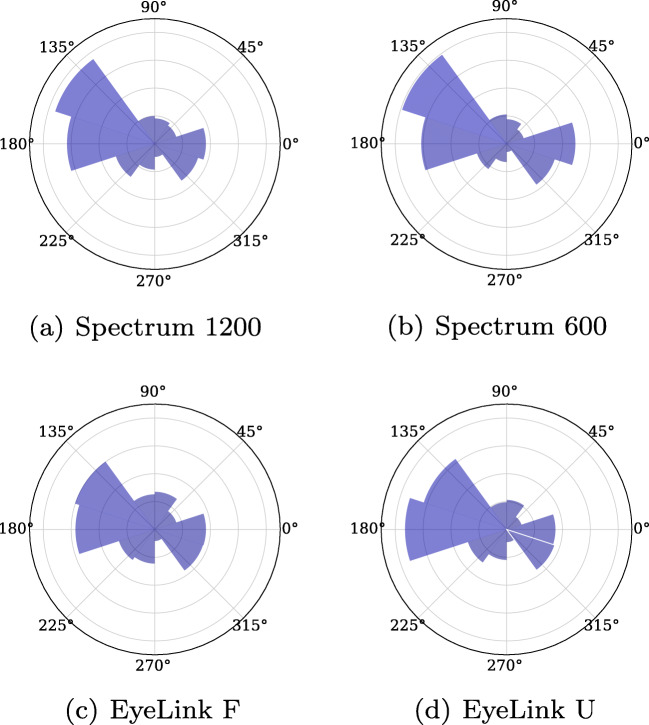
Angular histograms for microsaccade directions from all four participants from Experiment I. Zero (0) degrees represents microsaccades to the right, and 90 degrees upward microsaccades

Other frequently reported properties of microsaccades are their amplitudes and directions. Figure 6 shows both the (a) amplitude (maximum excursion during the microsaccadic interval) and (b) displacement (distance between onset and offset) of microsaccades and Fig. 7 illustrates microsaccade directions.

Again, there are only small differences across setups in both amplitude, displacement, and direction of the detected microsaccades. Note that the ranking of participants on amplitudes and displacements in Fig. 6 is stable across setups.

**Fig. 8:**
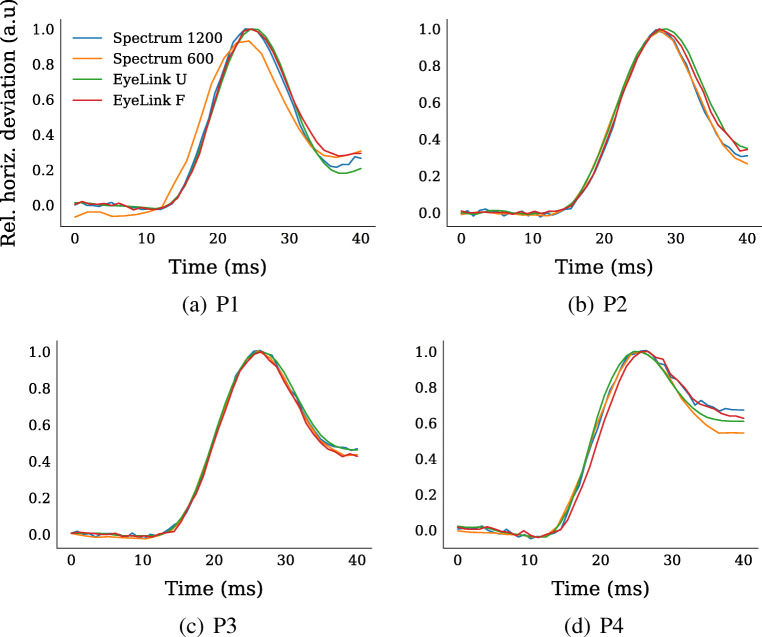
Microsaccade shapes across the recording setups for each participant (P1–P4, *left to right*). Each line represents a scaled average of horizontal gaze signals extracted for microsaccades with amplitudes around 0.2 deg

Finally, we align and plot the average waveform of a microsaccade for each recording setup and participant (Fig. 8). The waveforms were generated by extracting all microsaccades with amplitudes (*A*) in the range *A* = [0.14,0.26] deg, and then normalizing each waveform by its maximal value. As can be seen, the waveforms are virtually indistinguishable across different recording setups for all participants.

### Discussion

Eye-tracker data recorded from four experienced participants in a fixation task generated similar data quality and microsaccade rate across the tested setups. Besides the obviously higher precision in the EyeLink F setup, data from the four setups seem comparable.

## Experiment II

In early work, fixational eye movements were often recorded from experienced participants (Ditchburn & Foley-Fisher, 1967), sometimes the authors themselves, as was also the case in Experiment I of this paper. However, it is becoming increasingly more common to study microsaccades in naive or clinical populations (e.g., Alexander et al. 2018). It is therefore necessary to see if the results generalize to a wider, and less expert, population, which is the aim of Experiment II.

### Methods

The methods in Experiment II were the same as those of the first experiment with the following exceptions:

- A convenience sample of eight participants (30.8 ± 5.8 years, six female, two wore contact lenses, one wore glas ses) naive to the purpose of the experiment were recruited. None of them had long experience with eye tracking.
- To reduce potential variation in microsaccade rate across the recordings due to participants inventing their own tasks, they were asked to count silently at a pace of 1 Hz.
- The requirement to record blink-free segments was abandoned, making the experiment shorter and easier. The participants were asked to minimize the amount of blinking during the trials. Blink breaks between recordings were still included.

Also, since the trials could now include blinks, segments with potential blinks were identified in the pupil size signal (nan in the Spectrum data and values < 20 pupil area units in the EyeLink data), and 200 ms on each side of the missing data were omitted from further analysis to exclude artifacts associated with blink on-, and offsets. Since the Spectrum intermittently dropped single samples, these were replaced by means of linear interpolation prior to data exclusion, in order to prevent removing 400 ms of data in conjunction with a single sample loss.

### Results

#### Data quality

**Fig. 9:**
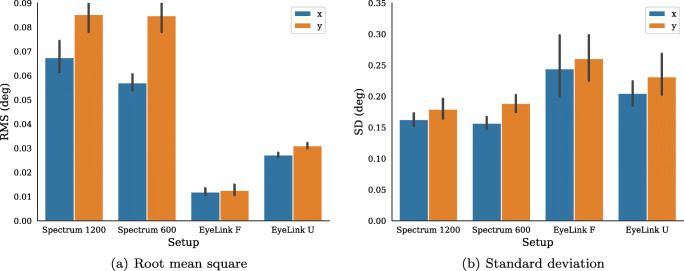
Experiment II. Root-mean-square (RMS) of inter-sample distances (**a**) and standard deviation (SD) (**b**) of gaze signals for each recording setup. *Error bars* show 95% confidence intervals of the mean; x and y refer to the horizontal and vertical dimensions of the data

Average accuracy over all validation points, participants, and eyes was (in degrees): EyeLink F (M = 0.39, SD = 0.44), EyeLink U (M = 0.35, SD = 0.20), Spectrum 1200 (M = 0.47, SD = 0.38, Spectrum 600 (M = 0.71, SD = 0.64). The precision of data recorded from novice participants was on average 0.05 ± 0.03 deg RMS and 0.20 ± 0.04 deg SD. These values were slightly higher than those recorded from the expert participants (RMS: 0.04 ± 0.02, SD: 0.19 ± 0.03). Precision results from the four setups are given in Fig. 9. Similarly to the expert participants, filtered EyeLink data provide the lowest RMS, followed by the EyeLink U, which has about a factor-two higher precision than the two Spectrum setups. The EyeLink setups provide on average the largest standard deviations.

**Fig. 10:**
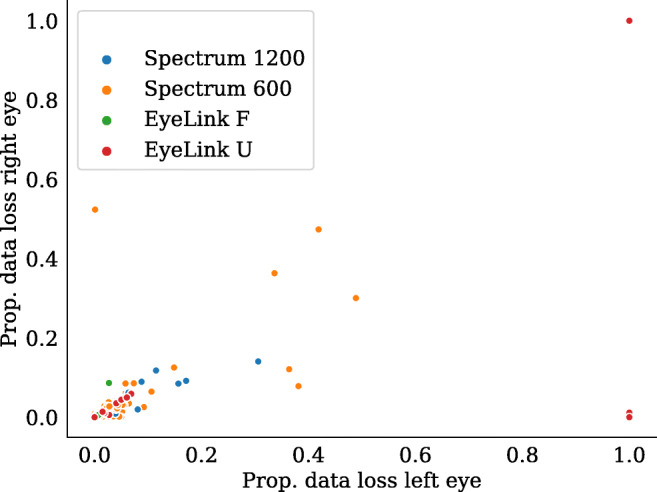
Proportion of data loss. Each *point* represents the proportion of data loss in the left and the right eye for each individual trial

The proportion of samples reported as missing (nan in the Spectrum and -32768 in the EyeLink) due to blinks and/or other recording problems was on average low (Spectrum 1200: 1.8 ± 1.2%, Spectrum 600: 4.2 ± 9.4%, EyeLink F: 0.6 ± 1.1%, EyeLink U: 4.5 ± 18.9%). Figure 10 shows the proportion of data loss for each participant and trial across the eye-tracker setups, separately for the left and the right eyes.

A low proportion of lost samples along the diagonal is likely indicative of blinks where both eyelids are closed at the same time, in a trial where most other samples were recorded successfully. Data points off the diagonal indicate that more samples were dropped in one eye compared to the other. As can be seen, the overall larger data loss in the EyeLink U compared to the EyeLink F setup can to a large degree be explained by the fact that five trials are missing all data either in both eyes (one trial) or in one eye (four trials). Inspection of these trials show that data were lost following blink breaks, where the eye tracker apparently failed to re-establish tracking after multiple blinks, or after an extended period of eyelid closure.

#### Microsaccades

**Fig. 11:**
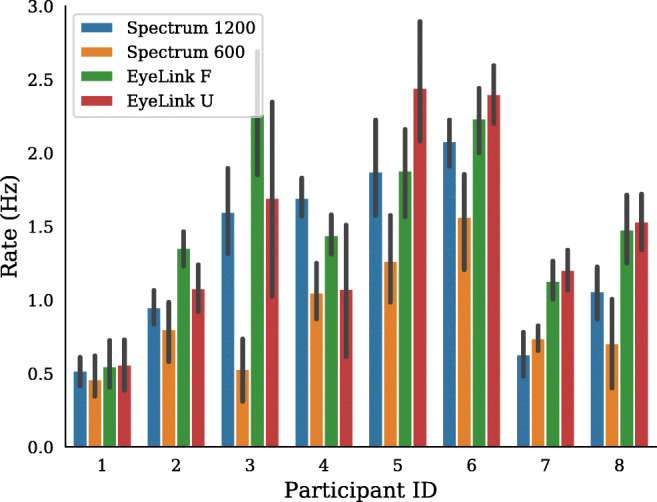
Experiment II. Microsaccades rates across the four setups for all participants. *Error bars* show 95% confidence intervals

On average, microsaccade rates ranged from 0.5 to 2.5 Hz across participants, which is in line with what has previously been reported in the literature (Martinez-Conde et al., 2009). Figure 11 shows microsaccade rate for each participant across the four setups. While it is difficult to immediately draw any conclusion from the plot, it appears that the microsaccade rate for the Spectrum 600 setup typically is lower compared to the other systems. This is particularly true for a few of the participants (P3, P5, P6).

**Fig. 12:**
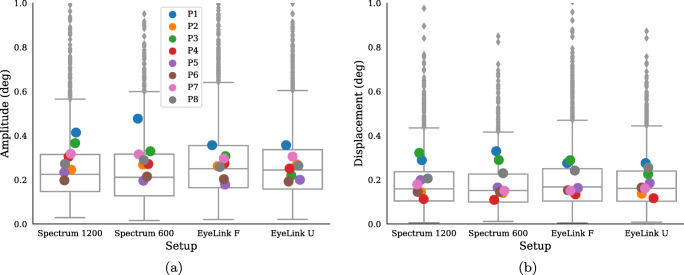
Microsaccades amplitude (**a**) and displacement (**b**) across the four setups for all participants in Experiment II. The *boxes* show the median value along with the upper and lower quartiles and the *whiskers* extend 1.5 times the inter-quartile range, and values outside this range are represented as *diamonds*. *Colored dots* represent each participant’s mean value

Microsaccade amplitudes (a) and displacements (b) are provided in Fig. 12. On average, there seem to be small differences in both amplitudes and displacements, and also large similarities for an individual across setups.

#### Discussion

Compared to Experiment I, Experiment II, using inexperienced participants, showed larger differences between the Spectrum setups and the EyeLink U setup in terms of RMS precision. On average, the RMS precision of the EyeLink U was about twice as high compared to the Spectrum setups. Fewer microsaccades seemed to be detected with data from the Spectrum 600 setup, in particular for a few participants. However, the extra 600 samples per second produced by the Spectrum 1200 apparently helped to pick up more microsaccades despite its slightly lower RMS precision (by 0.004 deg) compared to the Spectrum 600-Hz setup.

## Overall statistical analysis

The overall statistical analysis includes data from all participants in both experiments. All analyses were conducted with Python 3.6 and pandas (v. 0.23.4) as well as R (v. 3.6.1) and lme4 (v. 1.1.21) using participants as random effects with random intercepts. Post hoc pairwise comparisons across setups were conducted with the emmeans package (v. 1.4.1) after Bonferroni correction for multiple comparisons. Since amplitudes and displacements were highly correlated (*r* = 0.79, *p*< 0.001), only amplitude will be analyzed alongside microsaccade rate. Three questions related to data quality and microsaccades were addressed:

- Data quality
  - Are there statistical differences in precision (RMS and SD) across the setups?
- Microsaccades
  - Are there statistical differences in microsaccade rate and/or amplitude across the setups?
  - What is the test-retest reliability within and across setups?

The output of linear mixed effects models for RMS precision, amplitude, and rate is given in Table 1, where all setups are compared to the EyeLink F setup, represented by the Intercept in the model.

Unsurprisingly, the RMS precision of the EyeLink F was significantly higher than for all other setups. The RMS precision of the EyeLink U was significantly higher than the precision of both the Spectrum 1200 and Spectrum 600 setups (with 0.033 deg, *p* < 0.001 and 0.030 deg, *p* < 0.001, respectively). Pairwise comparisons revealed that the precision of the Spectrum 1200 was slightly lower (by 0.004 deg, *p* = 0.06) compared to the Spectrum 600 setup.

The EyeLink F setup had the largest SD, which was not significantly different from the EyeLink U setup (*p* = 0.16). Both EyeLink setups, however, had significantly larger SD compared to both Spectrum setups. The SD for the EyeLink U setup, for example, was about 0.04 deg (*p* < 0.001) larger compared to the Spectrum 1200 setup. The difference (0.001 deg) between the two Spectrum setups was non-significant (*p* = 1.00).

**Fig. 13:**
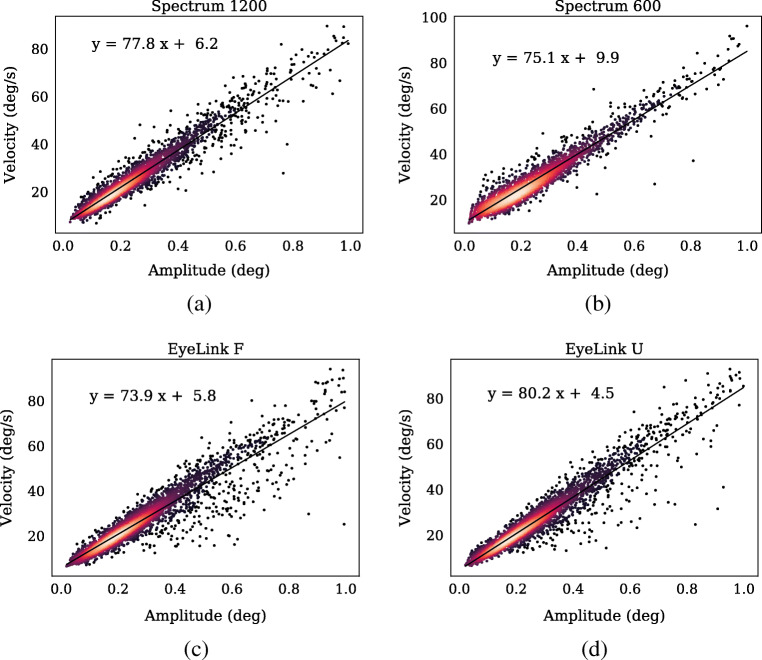
The main sequence for the four setups. The equation in the top left corner of each plot describes the parameters of a linear fit to the data, illustrated by the *solid line*. The color of data points is related to the point density, where warmer colors represent denser parts of the distribution. Each panel contains data from all participants in both experiments

Similar pairwise comparisons showed that the EyeLink U had significantly larger microsaccade amplitude compared to the Spectrum setups (Spectrum 1200: 0.033 deg lower, *p* < 0.001, Spectrum 600: 0.037 deg lower, *p* < 0.001). There was no significant difference between the two EyeLink setups (0.014 deg lower for the EyeLink F setup, *p* = 0.67). Since (micro)saccades are known to follow the ‘main sequence’, i.e., a systematic relationship between amplitude and peak velocity (Rolfs, 2009), we plot in Fig. 13 the main sequence of microsaccades in the four different setups. As can been seen, the majority of microsaccades are in close proximity with the linear fit to the data for each setup, verifying the expected relationship.

Finally, the Spectrum 600 had a significantly lower microsaccade rate compared to the other setups (EyeLink F: lower by 0.27 Hz, *p* < 0.001, EyeLink U: 0.34 Hz, *p* < 0.001, Spectrum 1200: 0.16 Hz, *p* = 0.02). The Spectrum 1200 setup had an on average 0.15 Hz lower microsaccade rate compared to the EyeLink setups, a difference which according to the pairwise comparisons was significant for the EyeLink U (0.19 Hz, *p* = 0.003), but not for the EyeLink F (0.12 Hz, *p* = 0.16).

Comparing the results obtained with a fixed *λ* = 6 to using an optimal *λ* value (Engbert & Mergenthaler, 2006) or the algorithm by Otero-Millan et al., (2014) provided similar overall results with respect to amplitude and rate (cf. Appendix D). The amplitudes were in general lower in the Spectrum setups compared to the EyeLink setups, but the differences were small (a few min of arc). Rates were most similar across the two EyeLink setups, and more similar between the EyeLink and Spectrum 1200 setups compared to the EyeLink and Spectrum 600 setups. To investigate whether the different algorithms identified similar microsaccades overlapping in time (*cf.* Appendix D), similarities between pairs of algorithms across setups are visualized in Fig. 17, and quantified with the *F*_1_-score, i.e., the harmonic mean of precision and recall. The plot shows that the agreement generally is high (close to 1), and that the *F*_1_-score is the highest for the EyeLink setups followed by the Spectrum 1200 and Spectrum 600 setups.

**Fig. 14:**
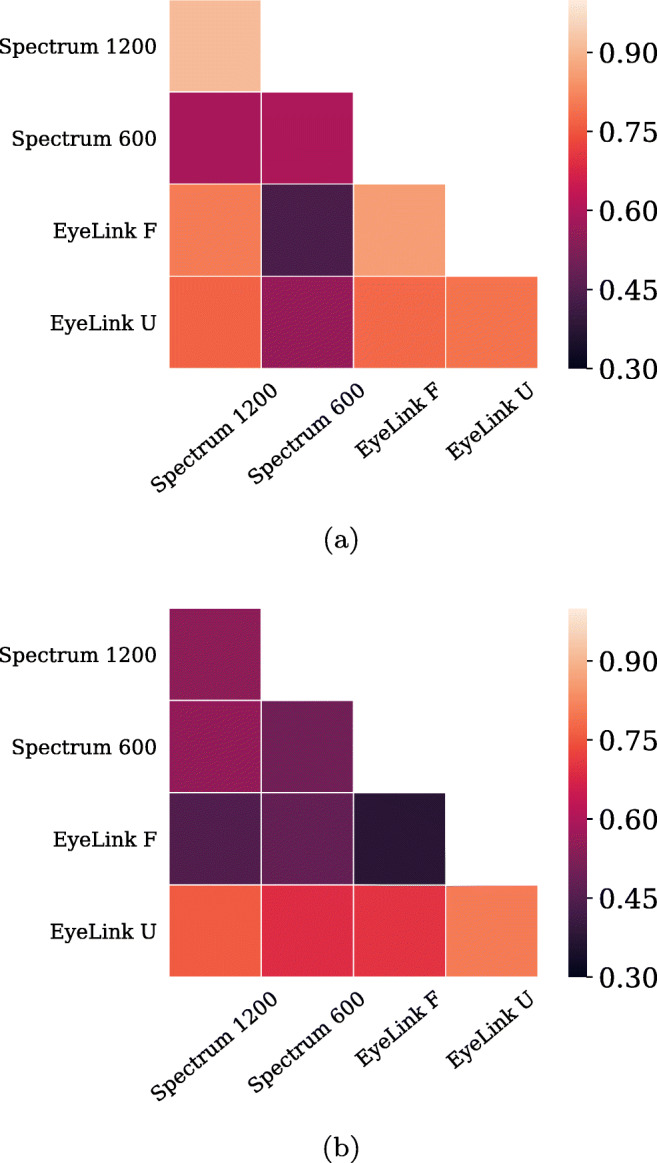
Test-retest correlation withing and across setups for microsaccade rate (**a**) and amplitude (**b**). The *numbers* correspond to the average values of comparing the first recording in one setup with the second recording in the other setup and vice versa

**Fig. 17:**
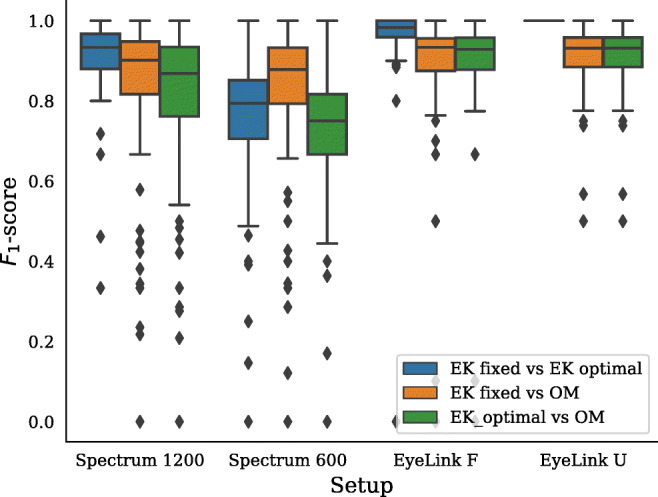
F_1_-scores for comparisons of three methods for microsaccade detection. An F_1_-score of 1 means that two algorithms fully agree on the classification, whereas 0 means no agreement

To quantify the test-retest reliability in microsaccade rate and amplitude, the Pearson correlation coefficient was computed within and between two repeated recordings of one (intra) or a pair (inter) of setups. Figure 14 illustrates the correlation between repeated recordings within and across setups for microsaccade rate (a) and amplitude (b), where brighter colors indicate a higher correlation. For microsaccade rate, the correlations within and between eye-tracker setups are generally high. Noteworthy is that the main diagonal, showing the intra-setup correlation, is not markedly higher than the inter-setup correlations, meaning that one can replace one system with another with small changes in microsaccade rate. The exception seems to be the Spectrum 600 setup, which has a low correlation in microsaccade rate both with itself and the other setups.

The test-retest reliability for microsaccade amplitude shows a more complex pattern, where it is difficult to discern any setup-specific trend, other than the EyeLink U appears to be more similar both to itself and the other setups.

## Discussion

We compared eye-tracker data quality and commonly used microsaccade parameters between the currently most used system in microsaccade research, the EyeLink 1000 Plus, and the Tobii Pro Spectrum. Since both systems can record in different modes, two recording setups for each system were used: 600 Hz and 1200 Hz for the Spectrum and filtered and unfiltered data with the EyeLink. The two highest sampling frequencies for the Spectrum were used to test whether the 1200-Hz mode provided noisier data compared to the 600-Hz mode, due to the likely shorter exposure time in the former case. Some microsaccade researchers record data with the proprietary heuristic filter turned on, therefore we compared filtered and unfiltered data. Two experiments were conducted to test the setups on different target groups: one with experienced eye-tracking experts (Experiment I) and one with inexperienced participants (Experiment II).

Overall, RMS precision, perhaps the most important property of an eye tracker intended for research on fixational eye movements, was about a factor two higher for unfiltered data (EyeLink U) compared to the Spectrum run in 600-Hz (Spectrum 600) and 1200-Hz (Spectrum 1200) modes. Unsurprisingly, EyeLink data recorded with the heuristic filters turned on (EyeLink F) generated the highest precision. Importantly, the filters do not seem to distort the recording of actual eye movements since microsaccadic waveforms appear unaffected and also since the power spectral density deviates from the other recording setups only at frequencies above 100 Hz, which is above the range of frequencies where information about oculomotor behavior is found (Findlay, 1971). At the same time, besides being proprietary and thus limiting reproducibility, the EyeLink filter does not seem to be critical for microsaccade detection, since a similar number of microsaccades is found in the EyeLink U setup, where these filters are switched off. The RMS precision of the Spectrum 1200 was slightly lower (0.004 deg) compared to the Spectrum 600. Since this difference in precision is very small, one would expect (as the results also show) that the two-fold increase in sampling frequency offered by the Spectrum 1200 Hz would make a net improvement compared to 600 Hz data in term of microsaccade detection. In theory, the higher RMS precision of the EyeLink U compared to the Spectrum setups should increase the signal-to-noise ratio, and therefore allow researchers to better distinguish very small microsaccades from noise. This could for instance be tested by co-recording the EyeLink and Spectrum setups with another system with an established high sensitivity to measure microsaccades, such as the dual Purkinje eye tracker (Crane and Steele, 1985) or scleral search coils (Collewijn, 1999; McCamy et al. 2013). Another way would be to rotate an artificial eye in very small steps, and see whether these steps are accurately reflected in the eye tracker signal (Reingold, 2014; Holmqvist & Blignaut, 2020).

The standard deviation (SD) was on average larger in the EyeLink compared to the Spectrum. One could only speculate about why this is the case. One hypothesis is that this is due to a relatively larger pupil size artifact in the EyeLink (Wyatt, 2010; Brisson et al., 2013; Drewes et al., 2014; Choe et al., 2015) compared to the Spectrum, causing drift in the gaze signal due to pupil size changes. However, whether the pupil size artifact is indeed smaller in the Spectrum remains to be tested. Another hypothesis is that gaze estimation in the Spectrum is less sensitive for very small head movements that participants make despite having their heads supported by a chin-, and forehead rest.

Considering the three different methods to identify microsaccades, the number of detected microsaccades did not change systematically across three of the setups (EyeLink F, EyeLink U, and Spectrum 1200) meaning that the setups could be used interchangeably when microsaccade rate is the main variable of interest. Data from the Spectrum 600, however, provided a significantly different rate of microsaccades as well as a poorer test-retest reliability. Thus, microsaccade researchers should use the highest available (1200 Hz) sampling frequency of the Spectrum. It should be noted, however, that the similarity in rate as well as the agreement in where different algorithms find microsaccades overall seem a bit higher when comparing the two EyeLink setups, compared to other combinations of setups. Whether this is related to the fact that the tested algorithms were mainly developed with EyeLink data remains to be investigated.

The comparison of microsaccade amplitudes were perhaps more difficult to interpret. In general, amplitudes recorded with the EyeLink setups were a bit higher (with about 0.02 deg). It is however known that amplitudes typically are overestimated in pupil-CR eye trackers like the EyeLink, since the pupil center moves relative the iris center during saccades (Kimmel et al., 2012; Nyström et al., 2013b; Hooge et al., 2016). Since we do not know how gaze is estimated by the Spectrum, we can only speculate whether it somehow is less affected by such relative pupil movements during saccades. Given the similarity of microsaccade trajectories across setups (Fig. 8), it seems likely that all setups are similarly influenced by relative pupil movements during microsaccades. Another potential explanation is that the Spectrum finds microsaccades with smaller amplitudes. This is however unlikely due to its higher noise level compared to the EyeLink setups. In terms of test-retest reliability of microsaccade amplitude, a complex pattern emerged likely indicating that microsaccade amplitudes varied across separate recordings, or that the eye-tracker setups or microsaccade detection algorithm are poor at estimating the amplitudes robustly.

There are more properties of an eye tracker that may be relevant to a researcher planning to investigate fixational eye movements such as microsaccades. First, although accuracy typically is not critical in most research of fixational eye movements, there are exceptions. Currently, high enough accuracies to allow gaze contingent experiments as conducted by e.g., Poletti et al., (2013), require a non-video-based eye tracker such as the dual Purkinje eye tracker (DPI). One reason that video-based eye trackers typically have poorer accuracy is due to the pupil size artifact described earlier. Second, the EyeLink and the Spectrum require different amount of skills by the operator of the eye tracker. While the Spectrum does not give the operator many ways to change the recording setup in case there is a problem to record a participant, the EyeLink is highly customizable and flexible, which often makes it possible to tweak the setup such that data can be acquired from almost any participant. Moreover, in the EyeLink, there is often a direct mapping to what can be seen in the eye image and why the tracking does or does not work. In the Spectrum, an eye image can look good (eye features clearly seen), but still the calibration fails for unknown reasons. However, for the beginning operator, all the flexibility offered by the EyeLink in combination with manual work (adjusting position of camera and illuminators, focus, and pupil and corneal reflection thresholds) may be overwhelming, and a system with few degrees of freedom where everything usually works automatically may be preferred. More research is required to make any definite claims about which system is better for microsaccade researchers in terms of usability and acquiring data with high quality from a large population.

In this paper, the participants’ heads were stabilized with a chin- and forehead rest. Another open question is whether the results would be different when participants are allowed to move their heads (see e.g., Skavenski et al. 1979). Both the Tobii Pro Spectrum and the EyeLink 1000 Plus offer head free recordings, so this could be tested in future work.

Finally, it should be noted that we cannot make any definite claims of whether the microsaccade rates and properties we report reflect the underlying eyeball rotation. Consequently, it is not known whether differences in microsaccade rate across the setups mean that data from one setup contain more ‘genuine’ microsaccades than another setup, or whether more episodes of the data were erroneously classified as microsaccades.

We conclude that the Tobii Pro Spectrum is a useful tool for microsaccades researchers, since it identifies microsaccades at similar rates and shapes as the current standard in the field: the EyeLink 1000 Plus. Although unfiltered data from the EyeLink 1000 Plus had about a factor two higher RMS precision compared to data acquired with the Spectrum, this did not translate into systematic differences in microsaccade properties. When using a Tobii Pro Spectrum for microsaccade research, we recommend researchers to use the Spectrum 1200 Hz, and not the 600 Hz, setup, to reduce the risk of missing microsaccades or mis-classifying noise as microsaccades.

## Figures and Tables

**Fig. 15:**
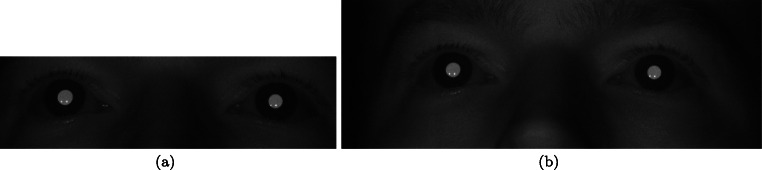
Eye images from the Tobii Pro Spectrum captured at (**a**) 1200 Hz (8 bit 384*x*105 pixels) and (**b**) 600 Hz (8 bit 495*x*175 pixels). From the bright appearance of the pupil, it is clear that this participant was recorded in bright pupil mode. Whether a participant should be recorded in bright or dark pupil mode is decided by the eye tracker during the calibration. Due to the likely shorter exposure time in the 1200 setup, it is possible that image quality, and hence precision, deteriorates

**Fig. 16:**
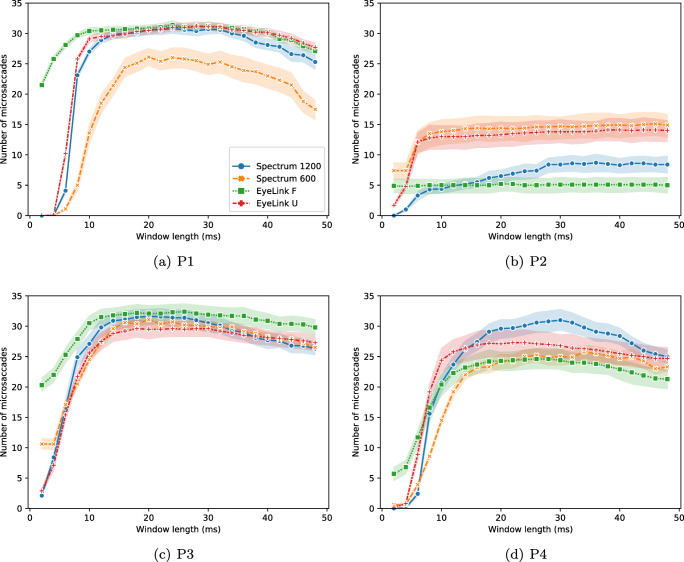
Influence on the number of detected microsaccades after applying a Bartlett window filter of a certain length to the gaze data before detecting microsaccades. Each subfigure represents one participant. *Error bands* show 50% confidence intervals

## Appendix A: Tobii Pro Spectrum eye images

Example eye images acquired from the Tobii Pro Spectrum at (a) 1200 Hz and (b) 600 Hz.

## Appendix B: Precision values

Tables 2 and 4 present precision (inter-sample RMS and SD) data for individual participants, recordings, and eye-tracker setups, in both dimensions (X, and Y).

**Table 2:** Experiment I: Precision values (RMS and SD) for each participant (pid), setup, recording (rec), and dimension (dim). Values represent visual angles in degrees

				RMS (deg)	SD (deg)
pid	Setup	rec	dim		
P1	EyeLink F	1	X	0.01	0.13
			Y	0.01	0.18
		2	X	0.01	0.14
			Y	0.01	0.16
	EyeLink U	1	X	0.03	0.13
			Y	0.03	0.15
		2	X	0.03	0.13
			Y	0.03	0.13
	Spectrum 1200	1	X	0.03	0.11
			Y	0.04	0.13
		2	X	0.03	0.11
			Y	0.04	0.11
	Spectrum 600	1	X	0.04	0.13
			Y	0.05	0.15
		2	X	0.04	0.11
			Y	0.05	0.12
P2	EyeLink F	1	X	0.01	0.18
			Y	0.02	0.16
		2	X	0.01	0.18
			Y	0.01	0.12
	EyeLink U	1	X	0.03	0.18
			Y	0.03	0.11
		2	X	0.03	0.15
			Y	0.03	0.10
	Spectrum 1200	1	X	0.04	0.12
			Y	0.04	0.12
		2	X	0.04	0.13
			Y	0.04	0.11
	Spectrum 600	1	X	0.03	0.13
			Y	0.03	0.12
		2	X	0.03	0.14
			Y	0.03	0.11
P3	EyeLink F	1	X	0.02	0.27
			Y	0.01	0.15
		2	X	0.01	0.32
			Y	0.01	0.22
	EyeLink U	1	X	0.06	0.27
			Y	0.05	0.18
		2	X	0.05	0.25
			Y	0.05	0.16
	Spectrum 1200	1	X	0.04	0.23
			Y	0.04	0.18
		2	X	0.05	0.27
			Y	0.05	0.16
	Spectrum 600	1	X	0.05	0.26
			Y	0.05	0.22
		2	X	0.05	0.24
			Y	0.05	0.18
P4	EyeLink F	1	X	0.01	0.32
			Y	0.02	0.47
		2	X	0.01	0.24
			Y	0.01	0.29
	EyeLink U	1	X	0.05	0.35
			Y	0.05	0.63
		2	X	0.05	0.29
			Y	0.06	0.34
	Spectrum 1200	1	X	0.05	0.22
			Y	0.06	0.18
		2	X	0.06	0.26
			Y	0.06	0.19
	Spectrum 600	1	X	0.05	0.17
			Y	0.06	0.16
		2	X	0.06	0.19
			Y	0.05	0.18

**Table 3:** Experiment II: Precision values (RMS and SD) for each participant (pid), setup, recording (rec) and dimension (dim). Values represent visual angles in degrees. Participants 1-4

				RMS	SD
pid	Eye tracker	recording	dim		
1	EyeLink F	1	X	0.01	0.19
			Y	0.01	0.24
		2	X	0.01	0.25
			Y	0.01	0.14
	EyeLink U	1	X	0.03	0.24
			Y	0.03	0.17
		2	X	0.03	0.16
			Y	0.03	0.16
	Spectrum 1200	1	X	0.07	0.15
			Y	0.08	0.15
		2	X	0.07	0.20
			Y	0.09	0.20
	Spectrum 600	1	X	0.05	0.14
			Y	0.06	0.13
		2	X	0.05	0.15
			Y	0.07	0.15
2	EyeLink F	1	X	0.01	0.83
			Y	0.01	0.26
		2	X	0.01	0.15
			Y	0.01	0.15
	EyeLink U	1	X	0.03	0.19
			Y	0.03	0.18
		2	X	0.03	0.15
			Y	0.03	0.10
	Spectrum 1200	1	X	0.04	0.14
			Y	0.06	0.15
		2	X	0.04	0.17
			Y	0.07	0.13
	Spectrum 600	1	X	0.04	0.13
			Y	0.06	0.16
		2	X	0.04	0.15
			Y	0.06	0.17
3	EyeLink F	1	X	0.01	0.14
			Y	0.01	0.23
			Y	0.01	0.59
	EyeLink U	1	X	0.02	0.13
			Y	0.02	0.23
		2	X	0.03	0.38
			Y	0.03	0.77
	Spectrum 1200	1	X	0.08	0.16
			Y	0.07	0.30
		2	X	0.07	0.14
			Y	0.07	0.17
	Spectrum 600	1	X	0.05	0.16
			Y	0.07	0.25
		2	X	0.05	0.24
			Y	0.08	0.19
4	EyeLink F	1	X	0.01	0.31
			Y	0.01	0.41
		2	X	0.01	0.23
			Y	0.01	0.13
	EyeLink U	1	X	0.03	0.22
			Y	0.03	0.29
		2	X	0.03	0.17
			Y	0.03	0.18
	Spectrum 1200	1	X	0.04	0.12
			Y	0.07	0.16
		2	X	0.09	0.22
			Y	0.09	0.21
	Spectrum 600	1	X	0.05	0.13
			Y	0.06	0.12
		2	X	0.05	0.12
			Y	0.07	0.13

**Table 4:** Experiment II: Precision values (RMS and SD) for each participant (pid), setup, recording (rec) and dimension (dim). Values represent visual angles in degrees. Participants 5–8

				rms	sd
pid	Eye tracker	recording	dim		
5	EyeLink F	1	X	0.01	0.36
			Y	0.01	0.21
		2	X	0.01	0.25
			Y	0.01	0.15
	EyeLink U	1	X	0.02	0.32
			Y	0.03	0.19
		2	X	0.02	0.15
			Y	0.03	0.23
	Spectrum 1200	1	X	0.05	0.10
			Y	0.05	0.13
		2	X	0.05	0.18
			Y	0.05	0.13
	Spectrum 600	1	X	0.05	0.12
			Y	0.06	0.14
		2	X	0.05	0.24
			Y	0.06	0.13
6	EyeLink F	1	X	0.01	0.24
			Y	0.01	0.11
		2	X	0.01	0.13
			Y	0.01	0.11
	EyeLink U	1	X	0.02	0.21
			Y	0.03	0.10
		2	X	0.02	0.14
			Y	0.03	0.11
	Spectrum 1200	1	X	0.04	0.13
			Y	0.06	0.10
		2	X	0.04	0.13
			Y	0.06	0.10
	Spectrum 600	1	X	0.04	0.10
			Y	0.05	0.08
		2	X	0.03	0.14
			Y	0.05	0.10
7	EyeLink F	1	X	0.01	0.19
			Y	0.01	0.37
		2	X	0.01	0.23
			Y	0.03	0.61
	EyeLink U	1	X	0.02	0.19
			Y	0.03	0.33
		2	X	0.02	0.19
			Y	0.03	0.23
	Spectrum 1200	1	X	0.06	0.22
			Y	0.08	0.23
		2	X	0.12	0.21
			Y	0.22	0.39
	Spectrum 600	1	X	0.09	0.24
			Y	0.09	0.30
		2	X	0.08	0.15
			Y	0.09	0.19
8	EyeLink F	1	X	0.03	0.19
			Y	0.04	0.22
		2	X	0.03	0.16
			Y	0.04	0.21
	EyeLink U	1	X	0.04	0.17
			Y	0.04	0.21
		2	X	0.04	0.17
			Y	0.04	0.18
	Spectrum 1200	1	X	0.09	0.16
			Y	0.09	0.17
		2	X	0.10	0.13
			Y	0.10	0.17
	Spectrum 600	1	X	0.07	0.16
			Y	0.13	0.23
		2	X	0.09	0.13
			Y	0.18	0.23

## Appendix C: Optimal length of lowpass filter for microsaccade detection

Feeding unfiltered 1000-Hz gaze data into the algorithm by Engbert and Kliegl (2003) and Engbert and Mergenthaler (2006) leads to missed detections of microsaccades due to too high noise levels (Nyström et al., 2017). Figure 16 shows how the number of microsaccades changes when applying a Bartlett window filter (Otero-Millan et al., 2014, cf.) of sizes 2 to 50 ms to the gaze data before applying the microsaccade detection algorithm.

Unsurprisingly, the already filtered EyeLink data (EyeLinkF) typically requires the shortest filter for the number of microsaccades to reach a relatively stable value. It can also be seen that the filter length required to reach this stable value is participant dependent, although filter lengths around 20 ms seem to produce a stable number of microsaccades for all participants and setups. This value (20 ms) is therefore selected as the filter length in the remainder of this paper.

## Appendix D: Comparison across microsaccade algorithms

To investigate the influence of the choice of detection algorithm on the results, three different methods were used to identify microsaccades: Engbert and Kliegl (2003) with a fixed *λ* threshold, Engbert and Kliegl (2003) with an optimal *λ* threshold based on surrogate data (Engbert & Mergenthaler, 2006), and Otero-Millan et al., (2014). Microsaccade detections for different algorithms were counted as overlapping, i.e., ‘hits’, if any samples overlapped in time. Based on data counting hits, misses, and false alarms, the *F*_1_ score was computed between all pairs of algorithms across all setups as described in Hooge et al., (2017). Figure 17 shows the *F*_1_-score between the output of the different algorithms. Overall, the comparisons have a high agreement (*F*_1_-score close to 1).

Tables 1, 5, and 6 show output from the linear mixed models using microsaccades from the three different methods for microsaccade detection.

**Table 5:** Output of the linear mixed effects models predicting microsaccade amplitude (Amp), and microsaccade rate (Rate) using output from the algorithm by Otero-Millan et al., (2014). The intercept corresponds to the EyeLink F setup

	Amp (deg)	Rate (Hz)
(Intercept)	0.35^∗∗∗^	1.36^∗∗∗^
	(0.03)	(0.17)
EyeLink U	− 0.00	0.05
	(0.02)	(0.07)
Spectrum 1200	− 0.05^∗∗^	− 0.23^∗∗∗^
	(0.02)	(0.07)
Spectrum 600	− 0.04^∗^	0.79^∗∗∗^
	(0.02)	(0.07)
AIC	− 498.08	777.49
BIC	− 473.24	802.40
Log Likelihood	255.04	− 382.74
Num. obs.	464	470
Num. groups: pid	12	12
Var: pid (Intercept)	0.01	0.31
Var: Residual	0.02	0.27

^∗∗∗^*p* < 0.001, ^∗∗^*p* < 0.01, ^∗^*p* < 0.05

**Table 6:** Output of the linear mixed effects models predicting microsaccade amplitude (Amp), and microsaccade rate (Rate) using output from the algorithm by Engbert and Kliegl (2003) using surrogate data to find optimal *λ* values (Engbert & Mergenthaler, 2006). The intercept corresponds to the EyeLink F setup

	Amp (deg)	Rate (Hz)
(Intercept)	0.29^∗∗∗^	1.37^∗∗∗^
	(0.02)	(0.16)
EyeLink U	0.01	0.13^∗^
	(0.01)	(0.06)
Spectrum 1200	− 0.04^∗∗∗^	0.14^∗^
	(0.01)	(0.05)
Spectrum 600	− 0.07^∗∗∗^	0.44^∗∗∗^
	(0.01)	(0.05)
AIC	− 1211.81	594.49
BIC	− 1186.85	619.44
Log Likelihood	611.90	− 291.24
Num. obs.	473	473
Num. groups: pid	12	12
Var: pid (Intercept)	0.00	0.29
Var: Residual	0.00	0.18

^∗∗∗^*p* < 0.001, ^∗∗^*p* < 0.01, ^∗^*p* < 0.05

## Footnotes

1 personal communication with Sam Hutton, SR Research, 2018-06-08.
